# Simultaneous Presentation of Hairy Cell Leukemia and Acute Lymphoblastic Leukemia

**DOI:** 10.4274/tjh.galenos.2021.2020.0503

**Published:** 2021-12-07

**Authors:** Mingyong Li, Yuan He, Kang Jiang, Juan Zhang

**Affiliations:** 1 Sichuan Academy of Medical Science, Sichuan Provincial People’s Hospital, Chengdu, China; 2 Shimian People’s Hospital, Ya’an, China

**Keywords:** Hairy cell leukemia, Acute lymphoblastic leukemia, Anemia

A 59-year-old man was admitted to the hospital because of repeated systemic bone pain for more than 1 month. His complete blood count revealed hemoglobin of 54 g/L, white blood cells of 12.35x10^9^/L, neutrophils of 0.247x10^9^/L, and a platelet count of 34x10^9^/L. Peripheral and bone marrow smears revealed cells with cytoplasmic projections (Figure 1, top left corner) and primitive cells (left upper diagonal line). These cells were negative for peroxidase staining (right bottom of the top left picture). Flow cytometry of marrow confirmed two clonal B-cell populations (top right): the first (red) was CD34^+^CD10^+^CD19^+^cCD79a^+^ (bottom left) and HLA-DR^+^D33^+^CD38^+^(dim)CD2^-^CD7^-^CD13^-^CD14^-^CD15^-^CD20^-^CD56^-^CD117^-^cIgM^-^cMPO^-^cCD3^-^ (not shown), diagnostic of B-lineage acute lymphoblastic leukemia (B-ALL). A second population (purplish-red) was CD103^+^CD11c^+^ (bottom right), CD19^+^CD25^+^CD123^+^CD22^+^CD20^+^sIgM^+^CD23^+^CD5^-^CD10^-^CD38^-^FMC7^-^slambda^-^ (not shown), and skappa light chain^+^ (bottom right), representing hairy cell leukemia (HCL). The BRAF V600E mutation was detected in the bone marrow aspirate sample. Therefore, the patient was diagnosed with simultaneous B-ALL and classic HCL. The association of ALL and HCL, either synchronous or metachronous, has rarely been reported [1]. In such cases, immunophenotyping with multiparameter flow cytometry is useful. This case highlights the indolent course of HCL, which can coexist with the acute process of ALL.

## Figures and Tables

**Figure 1:**
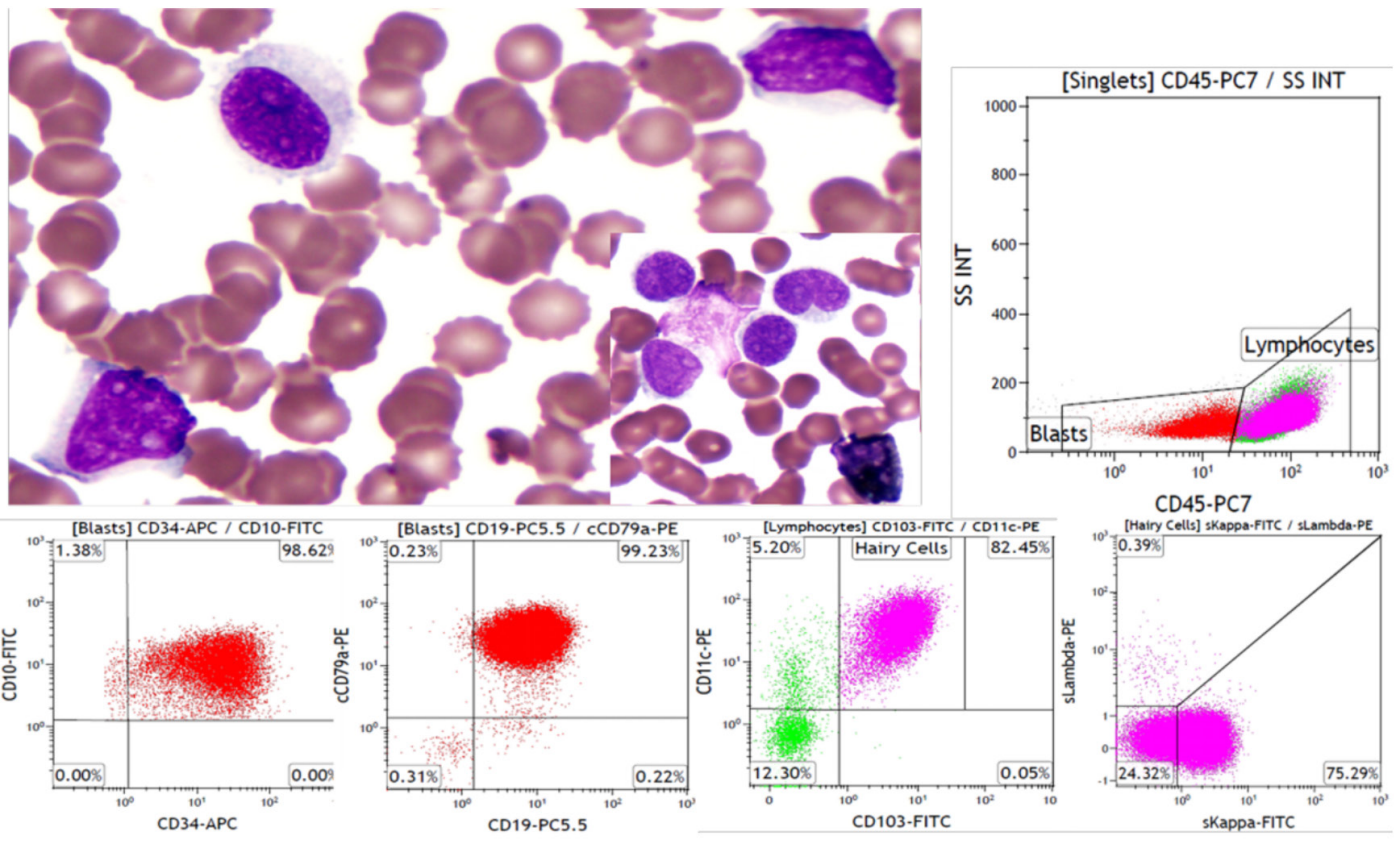
Peripheral and bone marrow smear revealed cells with cytoplasmic projections (top left corner, 1000^x^) and primitive cells (left upper diagonal line, 1000^x^). These cells were negative for peroxidase staining (right bottom of the top left picture, 1000^x^). Flow cytometry of the marrow confirmed two clonal B-cell populations (top right and bottom row; see text for details).
